# Analytical validation of bovine plasma ceruloplasmin measurement by *p*-phenylenediamine oxidation and effect of storage time and freezing temperature

**DOI:** 10.1186/s13028-017-0334-8

**Published:** 2017-10-04

**Authors:** Hussein Awad Hussein, Rudolf Staufenbiel

**Affiliations:** 1 Internal Veterinary Medicine, Department of Animal Medicine, Faculty of Veterinary Medicine, Assiut University, Assiut, 71526 Egypt; 2 Klinik für Klauentiere, Freie Universität Berlin, 14163 Berlin, Germany

**Keywords:** Biochemistry, Cows, Refrigeration, Thawing

## Abstract

**Background:**

Determination of ceruloplasmin (Cp) activity in plasma can provide an objective measure of the health of dairy cows as well as it can be used for various diagnostic purposes. The current study was designed to perform an analytical validation of a method for the determination of plasma Cp activity in dairy cows and to evaluate the influences of plasma storage times and temperatures as well as freeze–thaw cycles on the activity of this enzyme. This cohort was carried out on ten cows. For each cow, 24 aliquots of plasma, which were stored at different temperature regimes, were prepared. Both intra- and interassay coefficients of variation (CVs) were determined. The linearity was evaluated using bovine plasma Cp standard.

**Results:**

The mean values of intra- and interassay CVs were 1.08 and 2.12%, respectively. Results of linearity testing showed a high correlation coefficient (*r* = 0.998, *P* < 0.001). After 3 days of storage at room temperature and refrigeration, the plasma activity of Cp was significantly lowered (*P* < 0.05). Plasma samples kept at freezing for 3 months revealed insignificant changes in the activity of Cp. Repeated freeze–thaw cycles for three times had no significant influence on the activity of Cp.

**Conclusions:**

The method is easy and may be valid at values of Cp ranging from 100 to 1000 mg/L. It seems that keeping of plasma samples at room temperature and refrigeration longer than 3 days is unsuitable for Cp measurement. In addition, Cp remains stable in plasma samples stored at freezing as well as repeat freeze–thaw cycles.

## Background

Ceruloplasmin (Cp), a copper containing protein in plasma, was first identified in 1948 by Holmberg and Laurell [1]. It is a α2-glycoprotein synthesized in the liver containing six atoms of copper in its structure [2, 3]. In addition, Cp contains hexosamine, hexose, and neuraminic acid [4]. It has a ferroxidase activity and contains more than 95% of the copper present in plasma [5]. Cp has several functions. It is essential for iron homeostasis [6] and is involved in cellular prooxidant [7] and antioxidant [8] processes, as well as antibacterial host defense [9].

From the clinical point of view, Cp determination could provide information about the health status of cattle [10]. Cp is a mild to moderate acute phase protein that increases in concentration in association with inflammation [11–13]. Furthermore, it undergoes physiological changes during different lactation stages of dairy cows [14]. In addition, Cp has a potentiality for evaluation of copper status in dairy cows [15, 16].

Many quantitative methods had been used for Cp measurement in plasma or serum [4]. In veterinary clinical laboratories, most assays for estimation of Cp are based on oxidation of compounds such as *p*-phenylenediamine (PPD) or its *N*-dimethyl derivative and *O*-dianisidine dihydrochloride [17–19].

For multiple experimental time points, plasma samples may be subjected to different periods of storage before analysis [20]. From the practical point of view, the stored plasma samples may be reanalyzed to confirm the previous results or to perform additional analysis. Therefore, the stability of the analytes must be assured before giving results, or before establishing new investigations [21].

The objectives of the current study were to perform an analytical validation of a manual method for measurement of plasma Cp in Holstein dairy cows and to evaluate the influences of some methodological aspects related to plasma storage times and temperatures as well as freeze–thaw cycles on the activity of this enzyme.

## Methods

### Animals

Ten Holstein dairy cows (age range 3–6 years) at their mid lactation (15–18 weeks postpartum) were included in this study. All animals were between 2nd or 5th lactation seasons. All cows originated from a herd of State of Brandenburg, Germany, were housed in a free stall, and fed a diet of grass and maize silage and concentrate as a totally mixed ration (TMR). All cows were randomly chosen and were clinically healthy. On the basis of clinical examinations [22] made before sample collection, cows that had diseases which might have provoked an acute phase reaction were excluded from the study.

### Blood sampling and study design

**Fig. 1:**
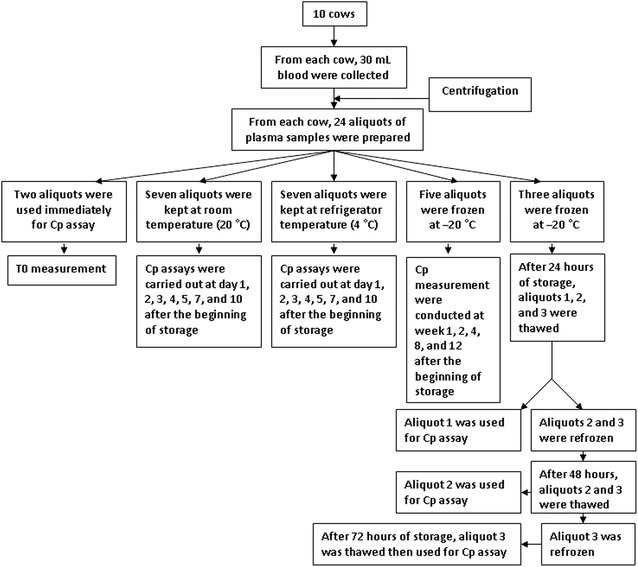
Schematic diagram representing study design and storage temperatures

From each cow, 30 mL of blood were sampled from a coccygeal vein into three heparinized vacutainer tubes. After centrifugation (3000 rpm, 15 min), plasma samples of each cow were harvested and pooled, and subsequently divided into aliquots. Twenty-four aliquots for each cow were prepared each aliquot containing 0.4 mL plasma. Two aliquots were used immediately for the assay (T0 measurements), and five and three aliquots were frozen at − 20 °C for studying the influence of freezing and repeated freeze–thaw cycles, respectively on the activity of Cp. Seven aliquots were kept at refrigerator temperature (4 °C) and the last seven aliquots were stored at room temperature (around 20 °C) (Fig. 1).

The three aliquots (1–3) that were kept at − 20 °C for studying the influence of freeze–thaw cycles on the activity of Cp were analyzed at three different days as the following: After 24 h, aliquots 1, 2, and 3 from each cow were thawed at room temperature (20 °C) for 30 min. Aliquot 1 was used for the assay (and then discarded), and aliquots 2 and 3 were refrozen. At 48 h, according to the same procedure, aliquot 2 was assayed and aliquot 3 was refrozen. At 72 h, aliquot 3 was thawed and assayed. The five aliquots from each cow stored at − 20 °C were analyzed after storage of 1, 2, 4, 8, and 12 weeks. The last 14 aliquots from each cow that were kept at room temperature and 4 °C were assayed at day 1, 2, 3, 4, 5, 7, and 10 after the beginning of storage.

### Biochemical assays

The activity of Cp was measured by *p*-phenylenediamine oxidation [14, 23]: 100 μL of plasma sample was diluted with 2 mL 0.1 M acetate buffer (adjusted to pH 5.45) and added to 1 mL freshly prepared buffered *p*-phenylenediamine solution (27.6 mmol/L) (Sigma-Aldrich, Germany) (adjusted to pH 5.45 using sodium bicarbonate), and then incubated at 37 °C. The absorbance reflecting the intensity of the purple coloured product was measured by a spectrometer at 530 nm, after 5 min (A5) and 30 min (A30) using sodium azide 1.5 M (Sigma-Aldrich, Germany) for stopping the enzyme reaction. The oxidase activity of Cp that was indicated by the absorbance was expressed in mg/L according to the following equation: Cp (mg/L) = (A30 − A5) × 752. The rate of formation of the coloured product of the enzymatic reaction is proportional to the concentration of plasma Cp; this is why the enzymatic activity is expressed as milligrams of Cp by liter of plasma instead of commonly used enzymatic units. There were 240 assays in total (10 cows, 24 aliquots, and 1 constituent).

### Analytical validation

#### Precision

Intra-assay coefficients of variations (CVs) were calculated after analysis of two plasma samples and analyzed ten times in a single assay run. Interassay coefficients of variations were determined by analyzing the same two samples in ten separate runs carried out on different days. All these samples were stored frozen at − 20 °C in aliquots.

#### Linearity

Linearity was evaluated directly by analyzing serial dilutions (1/2, 1/4, 1/8, 1/16, 1/32, 1/64, 1/128, and 1/256) of bovine plasma ceruloplasmin standard (BIO-VAR Ltd, Armenia). To determine the range of ceruloplasmin at which the relationship is linear, other dilutions (68, 61, 54, 47, 40, 33, 27, 20, 13, and 6%) of the dilution 1/4 of ceruloplasmin standard were prepared and analyzed. Furthermore, the limit of detection (LOD) of the assay was determined as the lowest concentration of an analyte (ceruloplasmin) in a sample that can be detected but not necessarily quantified, under the stated condition of the test [24]. In addition, the limit of quantitation (LOQ) was calculated as the lowest concentration of an analyte (Cp) in the sample that can be determined with acceptable precision and accuracy under the stated condition of the test [25]. Both LOD and LOQ were calculated from the linear regression using the following equation [24]:

$${\text{LOD}}\;{\text{or}}\;{\text{LOQ }} = \, \left( {{\text{F}} \times {\text{SD}}} \right) \, \div {\text{ b}}$$

where F is a factor of 3.3 in the case of LOD or a factor of 10 in the case of LOQ, SD: standard deviation of the intercept, b: slope of the regression line.

### Statistical analysis

Data were analyzed statistically using SPSS software (SPSS statistical program, SPSS Inc., Version 16, Chicago, IL, USA). Intra- and interassay CVs were calculated using routine descriptive statistical procedures. Ordinary regression analysis was performed to investigate the linearity under dilutions. The heteroscedasticity was tested using Breusch–Pagan test. Repeated measurements of the general linear model using Bonferroni multiple comparison test were used to investigate the influence of storage time on the plasma activity of Cp. Paired T test was used for statistical analysis of the differences between samples stored at room temperatures and refrigeration. For all statistical examinations, results were considered significant at *P* < 0.05. All data are presented as mean ± SD.

## Results

Table 1 shows the results of the precision study of Cp. Intra-assay CVs of plasma series 1 and 2 were 1.25 and 0.91%, respectively. In addition, interassay CVs of series 1 and 2 were 2.37 and 1.87%, respectively. It seemed that the between-run CVs were higher than the within-run, but all CVs were lower than 10%.

**Table 1:** Precision analysis of ceruloplasmin (Cp) using *p*-phenylenediamine assay

	Intra-assay			Interassay		
	Mean	Standard deviation	Coefficient of variation (%)	Mean	Standard deviation	Coefficient of variation (%)
Series 1 Cp (mg/L) (n = 10)	233	2.91	1.25	228	5.41	2.37
Series 2 Cp (mg/L) (n = 10)	276	2.50	0.91	275	5.15	1.87

**Fig. 2:**
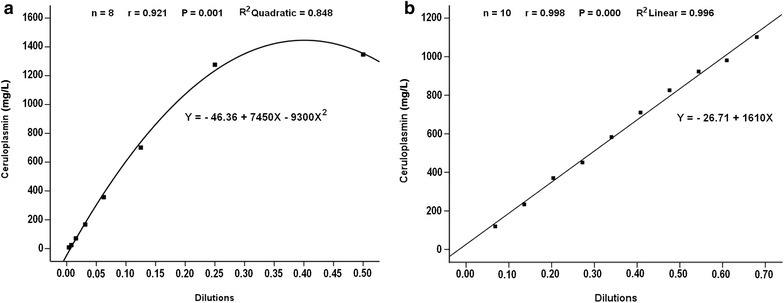
**a** Quadratic regression of ceruloplasmin standard. **b** Linear regression of diluted ceruloplasmin standard

**Fig. 3:**
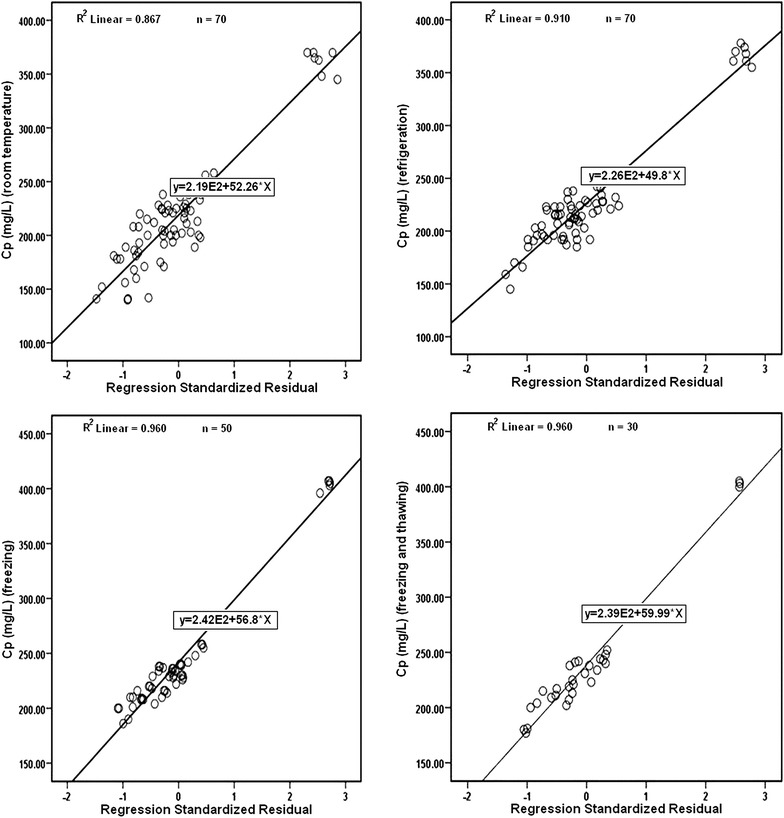
Linear regression between ceruloplasmin level (the dependent variable) and the regression standardized residuals at different storage temperatures

Figure 2 illustrates the relationships between Cp determined by the method and the different dilutions of Cp standard. Dilutions of the stock of Cp standard revealed quadratic regression equation with a correlation coefficient of *r* = 0.921 (Fig. 2a). A linear relationship was found using different dilutions of diluted Cp standard with a correlation coefficient of *r* = 0.998 (Fig. 2b). Linearity ranged from 100 to 1000 mg/L. Limit of detection (LOD) and limit of quantitation (LOQ) of Cp were 0.1 and 0.3 mg/L, respectively. The variance of residuals was constant with fitted values of the response variable. Furthermore, the residual analysis between the dependent variable (Cp, at different storage temperatures) and the regression of standardized residuals was linear and the distance from the fitted line and errors tended to be consistent (Fig. 3), indicating the residuals were homoscedastic.

**Fig. 4:**
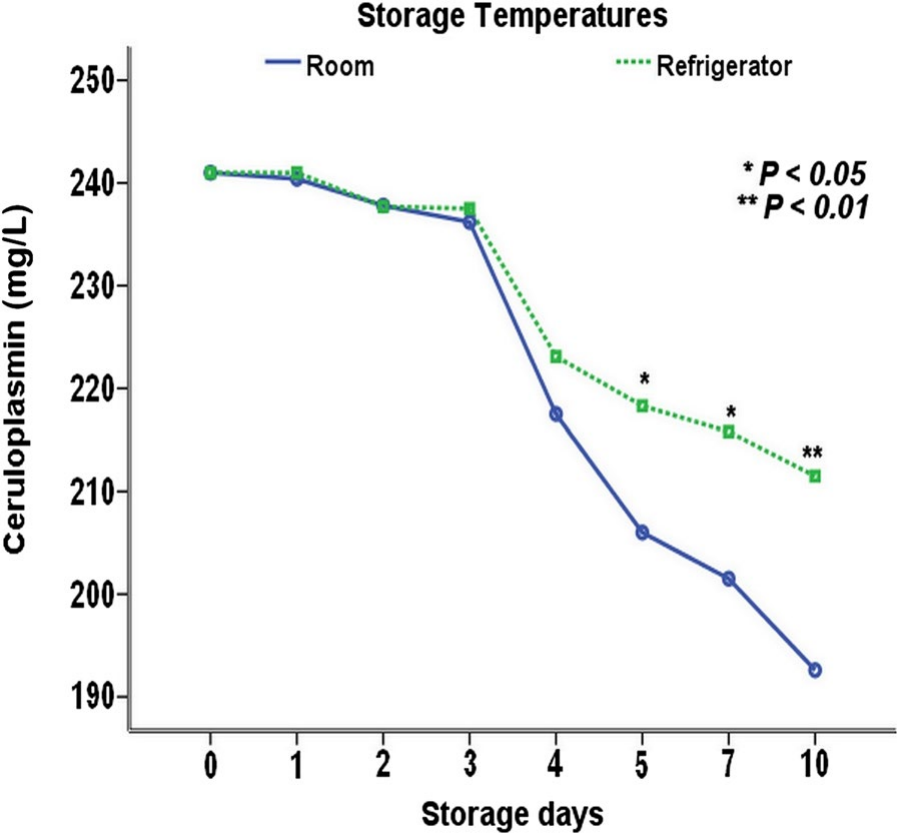
Influence of storage for 10 days at room temperature and refrigeration on the activity of ceruloplasmin

The plasma activity of Cp over time showed a highly significant decrease in samples stored at room temperature and at refrigeration at 4 °C (*P* < 0.001, Table 2). The plasma samples stored at room temperature and refrigeration had more markedly reduced Cp activity from day 4 onward, with the significantly lowest activity on day 10 of storage compared with the initial values (*P* < 0.05). Furthermore, on days 5, 7, and 10 of storage, the activity of Cp was significantly higher in samples stored at refrigeration than at room temperature (*P* < 0.05), (*P* < 0.05), and (*P* < 0.01), respectively (Fig. 4). In addition, Cp activity dropped by 20 and 12% on day 10 after plasma storage at room temperature and refrigeration, respectively.

**Table 2:** Influence of plasma storage at room and refrigeration temperatures on the activity of ceruloplasmin

	Storage temperature	Storage days							
		0	1	2	3	4	5	7	10
Ceruloplasmin (mg/L) (n = 10)	Room temperature (20–25 °C)	241 ± 59^a^	240 ± 50^a^	238 ± 51^a^	236 ± 47^a^	217 ± 57^b^	206 ± 62^bc^	201 ± 57^bc^	193 ± 57^c^
Ceruloplasmin (mg/L) (n = 10)	Refrigeration (+ 4 °C)	241 ± 59^a^	241 ± 50^a^	238 ± 49^a^	237 ± 51^a^	223 ± 52^b^	218 ± 56^bc^	216 ± 54^bc^	211 ± 55^c^

^abc^Values of different superscript letters in the same row differ significantly *P* < 0.05

For 3 months of plasma storage at freezing, the Bonferroni multiple-comparison test revealed insignificant changes (P > 0.05) in the activity of Cp (Table 3). On the other hand, three cycles of freezing and thawing had almost no effect on the activity of Cp (Table 4).

**Table 3:** Influence of plasma storage at deep-freezing on the activity of ceruloplasmin

	Storage temperature	Weeks of storage					
		0	1	2	4	8	12
Ceruloplasmin (mg/L) (n = 10)	Freezing (− 20 °C)	241 ± 59^a^	241 ± 59^a^	239 ± 61^a^	238 ± 57^a^	245 ± 59^a^	241 ± 59^a^

^a^No significant difference *P* > 0.05

**Table 4:** Influence of repeated freeze–thaw cycles on the activity of ceruloplasmin

	Freeze–thaw cycles			
	0	1	2	3
Ceruloplasmin (mg/L) (n = 10)	241 ± 59^a^	242 ± 61^a^	238 ± 62^a^	237 ± 61^a^

^a^No significant difference *P* > 0.05

## Discussion

Ceruloplasmin has been studied in dairy cows, and it is considered an important and useful indicator for evaluation of copper status in these animals [15, 16]. In addition, Cp has mild to moderate acute phase reaction in dairy cows with subclinical mastitis [26] and dairy calves with diarrhea [27]. No reports are available discussing the analytical validation or the influence of certain methodological aspects of the activity of Cp in dairy cows, but two studies have discussed the validation of automated methods for measuring this protein in canine serum [19] and pigs [28]. Therefore, it was of interest to perform an analytical validation of Cp assays as well as studying the influence of some methodological aspects, related to plasma storage times and temperatures, on the activity of this protein in Holstein dairy cows.

The results revealed a good assay precision with intra- and interassay CVs lower than 10%. These CVs were lower than those obtained using a manual method [29] or an automated method [19] for canine serum samples. Such variation in CVs may be attributed to the sample or animal species differences. As reported elsewhere [14], Cp activity in serum samples was approximately 30% lower relative to heparinized plasma.

The analytical accuracy was directly estimated by linearity assays. The linearity under dilution showed high regression coefficients, indicating that the assay measured Cp in a linear manner with a detection limit of 0.1 mg/L. These results were supported by previous studies in dogs [19] and pigs [28]. In the current study, the linearity ranged from 100 to 1000 mg/L, demonstrating that Cp correlated strongly with the dilutions; therefore, the method may give good results in this range.

Certain clinical trials and research studies may require delayed analyses of collected plasma samples. To accomplish these analyses, blood samples are separated by centrifugation, and then the harvested plasma is quickly frozen [30]. Furthermore, often researchers need to reanalyze a few or all of the plasma analytes to confirm conclusions or previous results. Under such circumstances, plasma samples may need to be frozen and thawed repeatedly. On the other hand, data on storage stability of Cp, and the effect of the temperature and duration of storage on its activity in veterinary medicine are still limited. Therefore, it is important to collect information about the effect of storage on its activity, as one of the pre-analytical factors that may affect the results of the assay.

The diminished activities of Cp in the plasma samples stored at room temperature and refrigeration may be related to the liability and degradation of this protein resulting from the changes in its molecular configuration during storage. Although the activities of Cp were lowered, its values were within the reference range (143–459 mg/L) as previously reported [14]. In previous studies, it was indicated that the stability of protein might be changed with the temperature storage [31, 32]. In the present study, the activity of Cp was higher in the plasma samples stored at refrigeration as compared to room temperature after 10 days of storage, indicating that the stability of Cp was higher at refrigeration than room temperature. In this study, it seems that storage of plasma samples at freezing for 3 months did not have a significant influence on the activity of Cp. Similarly, in another study, where human plasma samples were stored at − 20 °C over a period of 1 year, no significant changes in Cp activity was noted [33]. In the current study, three cycles of freezing and thawing had almost no effect on the activity of Cp, which may indicate a stable plasma Cp during repeated freeze–thaw cycles. However, the effect of repeated freeze–thaw cycles of plasma samples may need more than three cycles to be noticed, indicating further research work may be required in this regard.

## Conclusions

The evaluated method for Cp measurement could be successfully applied in Holstein dairy cows and may contribute to a wide use of Cp assays in bovine medicine. Furthermore, the method is easy, cheap, and may be valid at values of Cp ranging from 100 to 1000 mg/L. It seems that keeping of plasma samples at room temperature and refrigeration is unsuitable for Cp measurement. In addition, Cp remains stable in plasma samples stored at freezing as well as in samples subjected to repeated freeze–thaw cycles.

## Abbreviations

Cp: ceruloplasmin
CV: confidence intervals
LOD: limit of detection
LOQ: limit of quantification
PPD: *p*-phenylenediamine
SPSS: statistical package for the social sciences
TMR: totally mixed ration
